# Neural and behavioral effects of parent training on emotion recognition in mothers rearing children with attention-deficit/hyperactivity disorder

**DOI:** 10.1007/s11682-023-00771-9

**Published:** 2023-04-20

**Authors:** Kai Makita, Akiko Yao, Koji Shimada, Ryoko Kasaba, Takashi X. Fujisawa, Yoshifumi Mizuno, Akemi Tomoda

**Affiliations:** 1 Research Center for Child Mental Development, University of Fukui, Fukui, Japan; 2 Division of Developmental Higher Brain Functions, United Graduate School of Child Development, University of Fukui, Fukui, Japan; 3 Graduate School of Intercultural Studies, Kobe University, Kobe, Japan; 4 Biomedical Imaging Research Center, University of Fukui, Fukui, Japan; 5 Division of Affective and Cognitive Development, University of Fukui, Fukui, Japan; 6 Department of Child and Adolescent Psychological Medicine, University of Fukui Hospital, Fukui, Japan

**Keywords:** Attention-deficit/hyperactivity disorder, Emotion recognition, Magnetic resonance imaging, Parent training, Stress

## Abstract

This randomized controlled study examined neurological changes in socioemotional processing skills through parent training in caregivers of children with attention-deficit/hyperactivity disorder. Thirty mothers of children with attention-deficit/hyperactivity disorder were stratified into parent training and non-parent training groups. Functional magnetic resonance imaging was performed during the “Reading the Mind in the Eyes” test, and parenting difficulties were evaluated using the Parenting Stress Index and the Parenting Scale, twice (before and after parent training). Only mothers in the parent training group showed a significant decrease in Parenting Stress Index and Parenting Scale scores. They also demonstrated increased activity in the left occipital fusiform gyrus during the task of estimating emotions from facial pictures. We presumed that these changes might reflect the potential impact of enrollment in parent training in reducing stress, which might have increased activation of the fusiform gyrus.

**Supplementary information:**

The online version contains supplementary material available at 10.1007/s11682-023-00771-9.

## Introduction

Developmental disabilities in children and parenting difficulties are strongly related (Woodman et al., 2015). Attention-deficit/hyperactivity disorder (ADHD), one of the most commonly diagnosed neurodevelopmental disorders in childhood, has a relatively high incidence (approximately 5%) (American Psychiatric Association, 2013). Children with ADHD have functional problems in social and academic areas (Marshall et al., 2014). Additionally, ADHD symptoms in children can negatively affect their caregivers' psychological state, parenting behavior, and parent–child relationships (Hutchison et al., 2016; Pelham et al., 1998). These negative influences could result in inappropriate parental responses, such as maltreatment, creating risk factors for development of later conduct problems in the affected children (Chronis et al., 2007; Chronis-Tuscano et al., 2008).

Parent training (PT) is a psychosocial, support intervention that promotes a nurturing environment for childcare (parental understanding and response), which is widely known for its effectiveness (Daley et al., 2014) and is recommended for parents of children with ADHD (Posner et al., 2020). The PT program for ADHD includes essential information for parents on the characteristics of the disability and treatment options (Iwasaka, 2012; Zwi et al., 2011). Studies of PT in mothers rearing ADHD children have reported that PT significantly decreased their stress and improved their parenting self-efficacy and style (reduced overly harsh responses, lack of consistency, and ineffective limit-setting) (Abikoff et al., 2015; Heath et al., 2015; Shimabukuro et al., 2017). This reduced and prevented behavioral problems in children with ADHD (Smith et al., 2008; Tully et al., 2004). However, these previous studies focused on questionnaire data, which are prone to subjective bias. Few studies have examined the effectiveness of PT using more objective measurements (e.g., neuroimaging data).

A previous neuroimaging study reported that tasks that measure social skills and abilities, such as mind-reading tasks (“Reading the Mind in the Eyes test [RMET] (Baron-Cohen et al., 2001)), are negatively influenced by stress (Nolte et al., 2013) and may, therefore, be useful biomarkers of caregivers’ stress (Shimada et al., 2018). In the RMET, participants evaluate the affective states of others from static eye region images. Previous functional magnetic resonance (fMRI) studies have reported supplemental motor area, and inferior frontal, inferior temporal, fusiform, and occipital gyrus involvement during the RMET (Adams et al., 2010; Baron-Cohen et al., 2006; Castelli et al., 2010; Focquaert et al., 2010; Shimada et al., 2018). However, it is unclear whether improving caregivers’ stress by PT also alters activation of these areas.

Herein, we focused on the effects of PT on parenting stress and parenting practices in mothers of children with ADHD, at a neurological level. We hypothesized that PT would improve qualitative stress and unsound parenting practice indexes, as determined using parenting stress and discipline questionnaires. Moreover, we hypothesized that functional changes in maternal brain could be revealed by blood oxygenation level-dependent (BOLD) fMRI during the RMET. We expected that mothers enrolled in PT may respond more efficiently to others’ emotional status, and that this might be related to activity changes in brain regions involved in judging other’s affective states. Understanding the effects of PT at a functional neural level could potentially aid the development of more precise treatment strategies and biomarkers for evaluating treatment effects.

## Methods

### Participants

Thirty mothers (mean age 38.72 years; standard deviation [SD] 4.46 years) agreed to participate in this study. The inclusion criteria were as follows: mothers caring for ≥ 1 school-aged child (age 6–12 years) diagnosed with ADHD in a hospital or pediatric clinic. ADHD was diagnosed using the Diagnostic and Statistical Manual of Mental Disorders, Fifth Edition (American Psychiatric Association, 2013). The exclusion criteria were as follows: mothers who had participated in other PT programs within 2 months prior to enrolment, and changes in the child’s medication status during the study.

All participants had normal or corrected-to-normal vision, with no known medical, neurological, or psychiatric history (based on self-report questionnaires) and met the safety requirements for participation in an MRI study. Participants’ handedness was assessed using the Flinders Handedness Questionnaire (Nicholls et al., 2013; Okubo et al., 2014). The sex, medication status, and intelligence quotient (measured using the Wechsler Intelligence Scale for Children–Fourth Edition) of participants’ children were recorded.

The Ethics Committee of the University of Fukui approved the study protocol; all procedures were conducted in accordance with the Declaration of Helsinki. All participants provided written informed consent.

### Study design

Using a permuted-block randomization procedure for computer-based random number generation, mothers were randomly allocated to two groups: those who attended a 13-week PT course (PT group), and those who did not attend the course until after the study ended (non-PT group). In the PT group, mothers were taught and trained on the following contents in four major elements, by two trained clinical psychologists, throughout the 13-week course: 1) psychological/medical knowledge of ADHD and stress management skills, 2) how to observe and appropriately respond to the child’s behaviors and modify parenting skills accordingly, 3) how to provide clear explanation and rules for their children regarding their ADHD symptoms, and 4) effective methods for responding appropriately to the children’s non-adaptive behaviors stemming from their ADHD symptoms (see Supplementary Material 1, Parent training).

**Fig. 1:**
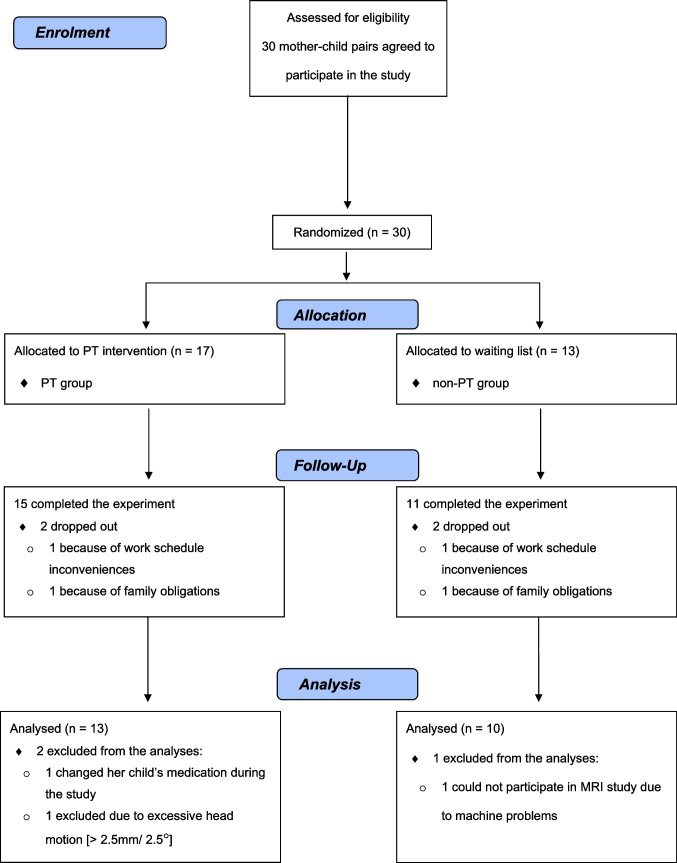
Flow diagram of this study. Abbreviations: Parent training (PT) group, mothers enrolled in the parent training intervention; non-PT group, mothers who were not enrolled in parent training intervention until after the end of the study

Before and after PT (approximately 90 days apart), mothers in both groups participated in the experiments at baseline (Time 1) and post-baseline (Time 2). Participants’ psychological characteristics were measured using psychological questionnaires, and their brain activities during emotion/gender judgement tasks were examined using fMRI. Of the 30 mother–child dyads (17 dyads in the PT group and 13 dyads in the non-PT group), seven (4 dyads in the PT group, and 3 dyads in the non-PT group) did not participate in the post-baseline session (4 dropped out and 3 were excluded from the analysis). Thus, the efficacy analysis included 13 dyads who received PT and 10 dyads who did not (for detail, see Fig. 1).

### Questionnaires for psychological testing

The Interpersonal Reactivity Index (IRI) (Davis, 1983; Sakurai, 1988), which comprises four subscales (empathic concern, personal distress, perspective taking, and fantasy), was used to measure the mothers’ empathic abilities. The State–Trait Anxiety Inventory (STAI) (Hidano et al., 2000; Spielberger et al., 1983) was used to measure their state and trait anxiety. The Beck Depression Inventory-II (BDI-II) (Beck et al., 1996; Kojima et al., 2002) was used to measure the mothers’ current depression. To measure maternal parenting stress, we used the Parent Stress Index (PSI) (Abidin, 1995; Narama et al., 1999), which includes child and parent stressor domains. Higher scores indicated that the rater scored the child’s behavior more negatively or that the rater assessed themselves or the parenting surrounding environment negatively. The Parenting Scale (PS) (Arnold et al., 1993; Itani, 2010) was used to examine the mothers’ parenting approaches in terms of laxness and overreactivity. This refers to the tendency to respond permissively (by passive and inconsistent parenting practices) or harshly (by overly authoritarian parenting practices), respectively, to child misbehavior. During the study, the BDI-II, PSI, and PS scores were measured twice (Time 1 and Time 2) to assess PT effects.

### Task and procedures

In the MRI study, mothers completed the revised RMET version (Baron-Cohen et al., 2001; Shimada et al., 2018), which consisted of two conditions. In one condition, the participant was presented 36 black and white photographs showing the eye region of a human face, with two words placed below the photo, one of which correctly identified the person’s emotion (the Theory of Mind [“ToM”] condition). In the other condition, the same 36 photographs were presented with the words “male” or “female” placed beneath, one of which correctly identified the person’s gender (the “GeN” condition). Participants were asked to indicate the word that they felt best described the person in the photograph by pressing one of two buttons in the scanner (Current Design, Philadelphia, PA). The task was run using Presentation software (Neurobehavioral Systems, Albany, CA), and projected using an MRI-compatible LCD display (BOLDscreen, Cambridge Research System, Cambridge, UK) placed on the back of the scanner. The participants viewed the stimuli via a mirror attached to the head coil.

The scanning was divided into two runs, each of which comprised a blocked CACBCA…design, where A: ToM condition, B: GeN condition, and C: resting condition. Both A and B lasted for 30 s, with the instruction word presented for 2.5 s. Then, six photographs were presented for 4.5 s each, followed by a 5-s inter-stimulus interval. C lasted for 15 s, during which only a fixation cross was presented. Each run contained 18 photographs of both A and B. The order of photographs in blocks A and B were counterbalanced across participants. Participants underwent training sessions before fMRI, outside the scanner, using training items (Supplementary Material 2 Figure S1).

### fMRI acquisition and analyses

A 3-T MRI scanner (Signa PET/MR, ver. 26, GE Healthcare, Milwaukee, WI) with a standard 8-channel head coil was used. Functional images were corrected with a T2*-weighted gradient-echo echo-planar imaging sequence to produce 40 whole-brain, continuous, transaxial slices (slice thickness, 3.5 mm with a 0.5-mm gap, repetition time [TR], 2500 ms; echo time [TE], 25 ms; flip angle [FA], 80°; field-of-view [FOV], 192 mm; 64 × 64 matrix; voxel dimension, 3.0 × 3.0 mm). A T1-weighted anatomical scan was also acquired using a fast spoiled-gradient recalled imaging sequence (TR, 8.46 ms; TE, 3.24 ms; FA, 11°; FOV, 256 mm; 256 × 256 matrix; 172 slices; voxel dimension, 1.0 × 1.0 × 1.0 mm).

In image data processing, for MRI signal equilibrium, the first four volumes were discarded from the analysis. The remaining 240 images were pre-processed and analyzed using the Statistical Parametric Mapping software (SPM ver.12, Wellcome Trust Center for Neuroimaging, London, UK) implemented in MATLAB R2019b (MathWorks, Natick, USA). Following slice-timing correction and spatial realignment, T1-weighted anatomical images were co-registered to functional images and segmented using a new segmentation algorithm with the DARTEL technique (Ashburner, 2007). Functional images were then spatially normalized into the Montreal Neurological Institute template, re-sampled to a spatial resolution of 3 × 3 × 3 mm^3^, and spatially smoothed with a 6-mm full-width at half-maximum Gaussian kernel.

After pre-processing, we first performed individual analyses to identify task-related activity. In this analysis, a design matrix containing two regressors of interest (ToM and GeN conditions) was created. Each condition was modelled with a general linear model, by using a 30-s box-car function convolved with the canonical hemodynamic response function, high-pass filtered at 1/128 Hz to minimize low-frequency artefacts. Six motion parameters were also included as nuisance regressors. Then, two contrast images (ToM and GeN) were obtained for each participant.

For group analyses, the two contrast images corresponding to the ToM and GeN conditions generated in the individual analysis were chosen. To investigate the effect of PT, between- and within-group differences (PT/non-PT vs. [Time 1/Time 2]) were analyzed using repeated-measures analysis of variance (ANOVA) within the framework of the SPM flexible factorial design.

Due to the small sample size, the following analyses were conducted, based on previous studies (Baumeister et al., 2018, 2019). To depict regions showing the PT effect in the PT group, as compared to the non-PT group (regions showing a significant decrease and/or increase after the PT intervention period), the contrast of interest was set as follows: PT (Time 1 > Time 2) > non-PT (Time 1 > Time 2) and PT (Time 2 > Time 1) > non-PT (Time 2 > Time 1). These comparisons were performed separately for both ToM and GeN conditions. In addition to whole brain analyses, a region-of-interest (ROI) analysis was conducted using WFU PickAtlas (https://www.fmri.wfubmc.edu/downloads) with the Automated Anatomical Labelling Atlas. Based on the hypothesis, to create the ROI mask, the following regions were selected bilaterally and combined into one mask image: supplemental motor area, inferior frontal, inferior temporal, fusiform, and occipital gyrus. The number of activated voxels were computed in the ROI mask for each condition.

Subsequently, to identify the expected intervention effect in the PT group, we conducted a within-subject SPM one-way ANOVA for separate comparison of the activities within the PT and non-PT groups under both conditions. The statistical threshold was set at *p* < 0.005, uncorrected at the peak level, and at *p* < 0.05 at the cluster level, family-wise error-corrected for multiple comparisons over the whole brain. The Neuromorphometrics Atlas (http://www.neuromorphometrics.com/) was used to identify the anatomical localization of significant clusters.

### Questionnaires and behavioral task data analyses

Fisher’s exact tests and individual *t*-tests were performed to compare the clinical and demographic characteristics between groups. Questionnaire scores and behavioral performance during the RMET were compared using mixed-model repeated-measures ANOVAs with time as the within-subject factor (pre- and post-PT period: Time 1 & Time 2), and groups as the between-group factor (PT and non-PT). For the RMET performance, the intervention effects were assessed on reaction times (RTs) and correct answer rate (accuracy) separately for the ToM and GeN conditions. To investigate significant effects further, Bonferroni-corrected pair-wise comparisons were performed. Statistical analyses were performed using IBM SPSS version 24 (IBM Corp., Armonk, NY, USA).

## Results

### Demographics and assessment outcomes at Time 1

The groups were similar in terms of age; BDI-II, STAI, PSI, PS, and IRI scores; years of education; handedness; and children’s data at Time 1 (Table 1).

**Table 1:** Demographics and psychological questionnaire scores of the mothers and children in the PT and non-PT groups at baseline (Time 1)

	Mothers in PT group (n = 13)	Mothers in non-PT group (n = 10)	Statistics	
	Mean (SD)	Mean (SD)	*t*-test [t(21)]	p-value
Age (years)	38.5 (5.1)	40.1 (4.6)	- 0.79	0.44
BDI-II	11.6 (7.5)	9.6 (7.2)	0.65	0.52
STAI				
State	40.5 (3.8)	40.0 (6.2)	0.24	0.81
Trait	48.8 (3.1)	49.1 (3.2)	- 0.25	0.81
PSI				
Child domain	109.2 (9.1)	103.7 (12.4)	1.22	0.24
Parent domain	111.7 (21.2)	103.1 (21.0)	0.97	0.34
PS				
Laxness	22.7 (6.3)	21.6 (5.2)	0.44	0.66
Overreactivity	40.5 (11.5)	41.7 (9.6)	- 0.28	0.79
IRI				
Empathic concern	11.6 (2.1)	12.0 (2.3)	-0.42	0.68
Personal distress	12.6 (3.3)	11.9 (2.6)	0.56	0.58
Perspective taking	14.8 (2.0)	16.2 (2.5)	- 1.52	0.15
Fantasy	12.4 (3.4)	11.8 (2.3)	0.47	0.64
	n (%)	n (%)	Fisher’s exact test	p-value
Completed at least 12 years of education	13 (100)	10 (100)		n.a
Right handedness	12 (92)	8 (80)		0.56
	Children in PT group (n = 13)	Children in non-PT group (n = 10)		
	Mean (SD)	Mean (SD)	t-test [t(21)]	p-value
Age (years)	9.0 (1.7)	9.4 (1.6)	-0.79	0.44
WISC-IV FSIQ	99.7 (13.9)	97.0 (13.6)	0.48	0.64
	n (%)	n (%)	Fisher’s exact test	p-value
Male children	10 (77)	10 (100)		0.23
Under medication	8 (62)	7 (70)		1.0

PT, mothers enrolled in parent training intervention; non-PT, mothers who did not enroll in the parent training intervention until after the end of the study; BDI-II, Beck Depression Inventory-II; STAI, State–Trait Anxiety Inventory; PSI, Parenting Stress Index; PS, Parenting Scale; IRI, Interpersonal Reactivity Index; WISC-IV, Wechsler Intelligence Scale for Children–Fourth Edition; FSIQ, Full Scale Intelligence Quotient; SD, standard deviation

### Changes in questionnaire scores and RMET performance between Time 1 and Time 2

For the BDI-II scores, 2 × 2 ANOVAs (Time [Time 1, Time 2] × Group [PT, non-PT]) showed no significant main effects or interactions. For the PSI child-domain scores, there was a significant Time × Group interaction, while the post-hoc within-group *t*-test yielded significantly lower scores at Time 2 than at Time 1 (*p* = 0.003, 95% confidence interval [CI]: -14.38, -3.47) in the PT, but not in the non-PT group (*p* = 0.23, 95%CI: -2.52, 9.92). For PSI parent-domain scores, we also found a significant Time × Group interaction, with a significant decrease in the PT group (*p* = 0.042, 95%CI: -12.68, -0.24), and a trend towards a significant increase in the non-PT group (*p* = 0.053, 95%CI: -0.09, 14.09). For PS overreactivity scores, there was a significant Time × Group interaction, with a significant decrease in the PT (*p* = 0.009, 95%CI: -11.415, -1.816), but not in the non-PT group (*p* = 0.75, 95%CI: -4.57, -6.37). No significant main effects or interactions were found for laxness scores.

**Fig. 2:**
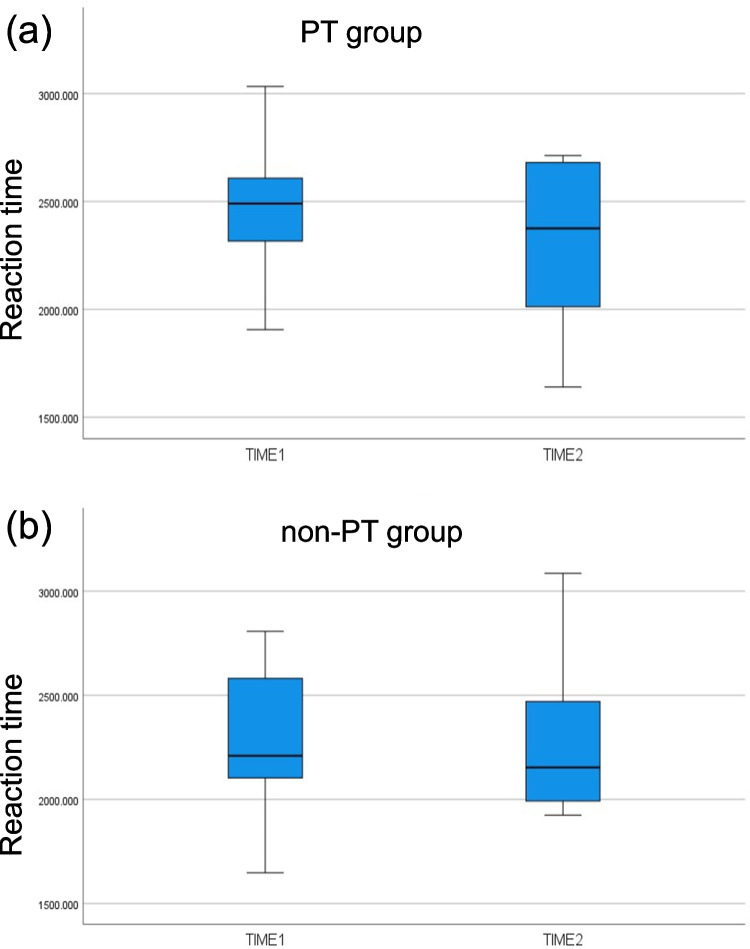
Box plot showing the reaction times under the ToM condition for both groups and at both time points. Reaction times at Time 1 and Time 2 for (**a**) PT group, and (**b**) non-PT group. Abbreviations: ToM, Theory of Mind; PT, mothers enrolled in parent training intervention; non-PT, mothers who did not enroll in parent training intervention until after the end of the study.

Under the ToM condition in the RMET, the RTs showed a trend toward significance for the main effect of Time (*p* = 0.058) and interaction of Time × Group (*p* = 0.050), but not for the main effect of Group (*p* = 0.538). A planned post-hoc *t*-test showed a significant RT decrease in the PT (*p* = 0.005, 95%CI: -313.93, -61.64), but not in the non-PT group (*p* = 0.96, 95%CI: -140.34, 147.31) (Fig. 2). For accuracy, there was a trend toward significance for the main effect of Time (*p* = 0.063), but not for the main effect of Group (*p* = 0.801) or interaction of Time × Group (*p* = 0.682). Other indices (RTs and accuracy under GeN condition) showed no significance or trend toward significance for the main effects or interactions (Table 2).

**Table 2:** Psychological questionnaire scores of outcome measures in the PT and non-PT groups at baseline (Time 1) and in the post-intervention period (Time 2)

	PT group (n = 13)			non-PT group (n = 10)			Statistics										
	Time 1	Time 2		Time 1	Time 2		ANOVA (Group main effect)				ANOVA (Time main effect)				ANOVA (Group × Time interaction)		
	Mean (SD)	Mean (SD)		Mean (SD)	Mean (SD)		F-value	p-value	Partial η^2^		F-value	p-value	Partial η^2^		F-value	p-value	Partial η^2^
BDI-II	11.62 (7.50)	8.23 (7.26)		9.6 (7.2)	9.10 (5.93)										1.08	0.310	0.09
PSI																	
Child-domain	109.15 (9.05)	100.23 (12.47)		103.70 (12.41)	107.40 (13.08)										10.08	0.005*	0.32
Parent-domain	111.69 (21.20)	105.23 (21.21)		103.10 (21.00)	110.10 (15.72)										8.80	0.007*	0.30
PS																	
Laxness	22.69 (6.29)	22.84 (7.67)		21.60 (5.19)	22.70 (5.87)										0.15	0.71	0.01
Overreactivity	40.46 (11.48)	33.85 (8.36)		41.70 (9.63)	42.60 (8.93)										4.61	0.044*	0.18
RMET																	
ToM																	
RTs	2474.61 (339.63)	2286.83 (372.36)		2288.29 (351.48)	2291.78 (384.43)		0.39	0.538	0.02		4.01	0.058	0.16		4.32	0.050	0.17
Accuracy	86.33 (5.37)	83.97 (7.45)		87.50 (6.18)	83.89 (5.52)		0.07	0.801	0.003		3.86	0.063	0.16		0.17	0.68	0.01
GeN																	
RTs	1191.53 (315.97)	1110.29 (191.48)		1077.19 (243.66)	1012.71 (264.62)		1.30	0.266	0.06		1.66	0.212	0.07		0.22	0.88	0.001
Accuracy	95.51 (4.0)	95.72 (3.87)		96.94 (3.06)	94.72 (4.43)		0.02	0.882	0.001		1.62	0.218	0.07		2.37	0.139	0.101

Accuracy, correct answer rate (%); PT group, mothers enrolled in the parent training intervention; non-PT group, mothers who were not enrolled in the parent training intervention until after the end of the study. Abbreviations: BDI-II, Beck Depression Inventory-II; GeN, gender judgement condition; PS, Parenting Scale; PSI, Parenting Stress Index; PT, parent training; RMET, Reading the Mind in the Eyes Test; RTs, Reaction Times (ms); ToM, Theory of Mind condition; SD, standard deviation. *p < 0.05

Furthermore, additional ANOVA for correct item RTs under the ToM condition showed a similar trend as above; the RTs showed a significant main effect of Time (*p* = 0.008) and a trend toward a significant interaction of Time × Group (*p* = 0.052). A post-hoc *t*-test for each group showed a significant decrease in RTs in the PT (*p* = 0.001, 95%CI: -323.96, -94.39), but not in the non-PT group (*p* = 0.56, 95%CI: -93.84, 167.91). However, ANOVA for incorrect items under the ToM condition revealed neither significant interaction (*p* = 0.074), nor significant within-group changes by post-hoc *t*-tests in the PT (*p* = 0.241, 95%CI: -470.33, 125.25) and non-PT groups (*p* = 0.163, 95%CI: -109.60, 606.19) (see Supplementary Material 3 for full results of 2 × 2 ANOVAs [Time (Time 1, Time 2) × Group (PT, non-PT)] conducted for questionnaire scores and RMET performance between Time 1 and Time 2).

### Imaging results

For the whole brain analysis of the repeated-measures ANOVA revealed no significant changes in activity (interaction contrasts) in any region in the PT group as compared to the non-PT group at Time 2, under the ToM or GeN conditions. Likewise, no significant results were shown in the additional ROI analysis.

As few studies have investigated the PT effect on brain function of mothers caring for children with ADHD, and to balance Type I and II error risks, we explored the potential PT effects by using more lenient threshold (*p* < 0.005, uncorrected, with a cluster size of k ≥ 40 voxels) (Lieberman & Cunningham, 2009; Nishiyama et al., 2015) in repeated-measures ANOVA. We then observed bilateral clusters of increased activation in the calcarine cortex, left inferior temporal gyrus, and right inferior occipital gyrus (Supplementary Material 4, Table S1). The ROI analysis revealed activity changes in the left calcarine cortex (including occipital fusiform gyrus) in the PT group as compared to the non-PT group at Time 2 (Supplementary Material 5, Table S2).

**Fig. 3:**
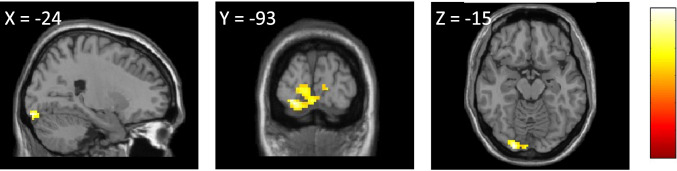
Regions showing significant differences between Time 1 and Time 2 in the PT group during the RMET. The colored regions show significantly increased activation after PT intervention in the PT group. The statistical threshold was set at *p* < 0.005, uncorrected at the peak level, and *p* < 0.05, family-wise error-corrected for multiple comparisons at the cluster level. Locations were defined using the Neuromorphometrics Atlas implemented in SPM. The color gradient bar on the right side of the figure indicates t-value, ranging from red (minimum) to yellow (maximum). Abbreviations: PT group, mothers enrolled in the parent training intervention. PT, parent training; RMET, Reading the Mind in the Eyes Test. X Y Z, Montreal Neurologic Institute coordinates.

Furthermore, planned within-group comparisons showed significantly increased activation in the left occipital fusiform gyrus in the PT group during the ToM condition at Time 2 as compared to Time 1 (Fig. 3, Table 3). The non-PT group showed no such difference. Other tests did not show any significant differences.

**Table 3:** Regions showing significant differences between Time 1 and Time 2 in the PT group (within-group comparisons)

Anatomical region	Side	MNI coordinates			Cluster	Voxel	Cluster
		x	y	z	Size	T value	P value
ToM condition: Time 1 vs Time 2							
Time 1 > Time 2							
[No significant activations]							
Time 2 > Time 1							
Occipital Fusiform Gyrus	Left	-24	-93	-15	193	5.43	0.028
GeN condition: Time 1 vs Time 2							
Time 1 > Time 2							
[No significant activations]							
Time 2 > Time 1							
[No significant activations]							

The threshold was set at *p* < 0.005, uncorrected at the peak level and *p* < 0.05, family-wise error-corrected for multiple comparisons at the cluster level. Locations were defined using the SPM Neuromorphometrics Atlas Abbreviations: GeN, Gender; MNI, Montreal Neurologic Institute; PT, parent training; ToM, Theory of Mind

Additionally, associations between increased signal changes of the left occipital fusiform gyrus, and degree of reduction of RTs under the ToM condition and reduced PSI and PS (Time 1 vs Time 2) scores were investigated using the MarsBar Toolbox (http://www.sourceforge.net/projects/marsbar). However, there were no significant correlations of brain activity with RTs or questionnaire scores (Supplementary Material 6, Table S3).

## Discussion

In our study, mothers enrolled in the PT program exhibited less parenting stress, as measured in both the child- and parent-domains of the PSI. PS scores revealed that mothers in the PT group adopted fewer inappropriate overreactive parenting practices.

Our findings were consistent with those of previous studies using PT for mothers with ADHD children, showing decreased maternal stress and enhanced positive parenting practices after PT (Chronis-Tuscano et al., 2013; Heath et al., 2015; Shimabukuro et al., 2017; Treacy et al., 2005). Presumably, these results indicate that PT promotes mothers’ understanding of the behavior of their children with ADHD, thus reducing their stress and improving parenting disciplines. Considering the content of PT, reduced stress might suggest that, by participating in PT, mothers increased their ADHD knowledge and their understanding of the cause-and-effect relationships between their own emotional responses to the child’s misbehaviors, which changed their perception of these behaviors, and thus reduced stress. For improved parenting practices, mothers might well learn the content and purpose of our PT; particularly training on how to provide clear explanations and rules for their children, and to respond appropriately to the children’s ADHD-related non-adaptive behaviors.

Additionally, we conducted PT as a group-based program. Previous studies have shown that group-based programs improved parenting stress and practices in mothers of children with ADHD (Shimabukuro et al., 2017; Treacy et al., 2005). The group-based program provides opportunities for mothers to discuss and share their concerns with peers having similar difficulties (in particular, ADHD), which may further motivate them to participate in the program and enhance their mental health. However, since the non-PT group was not given this opportunity, the effects may include not only those of PT itself, but also the effects of the group meetings.

**Fig. 4:**
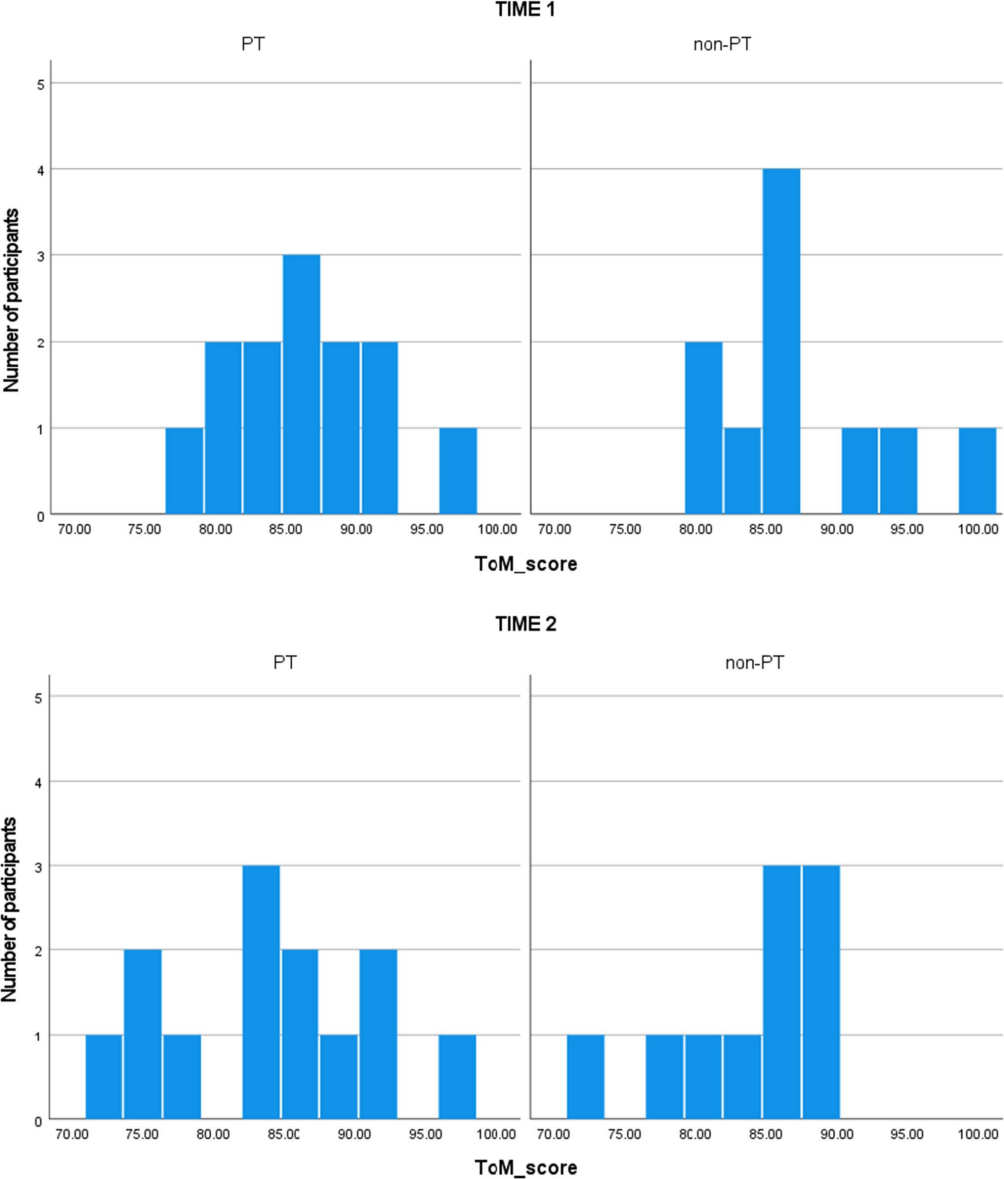
Histograms of accuracy (correct %) under the ToM condition at Time 1 and Time 2. Histograms of accuracy for ToM condition. Upper panel denotes scores in Time 1, and lower panel denotes scores in Time 2. Abbreviations: ToM, Theory of Mind; PT, mothers enrolled in parent training intervention; non-PT, mothers who did not enroll in the parent training intervention

For the behavioral performance of RTs in the RMET under the ToM condition, the main effect of Time and interaction of Time × Group showed a trend toward significance. The post-hoc test showed significantly reduced RTs at Time 2 only for the PT group. The results of the interaction of Time × Group and the post-hoc test suggest that the PT group tends to improve more with respect to RT compared to non-PT group. However, this should be interpreted with caution, as the main effect of Time showed that improvement tends to be time dependent regardless of the group. In additional analyses, the interaction effect on correct responses remained a trend toward significance, but the main effect of Time was (more strongly) significant, suggesting that the effect of time might be stronger than the effect of PT on the decrease in RTs to correct responses. For the behavioral performance of accuracy in the RMET, a trend toward significance was observed for the main effect of Time, showing somewhat worse performance at Time 2 regardless of the group. It is unclear why RTs and accuracy tended to decrease regardless of the group, but one possible mechanism may be reduced engagement with the task at Time 2 due to boredom and/or habituation (Orr & Stern, 1970). However, this study did not assess the degree of engagement with the task, and this interpretation remains a speculation. Regarding RMET scores, another possibility is the ceiling effect in neurotypical adults, and thus, this tool may be insensitive to slight differences (Black, 2019). Figure 4 shows the distribution of accuracy on ToM condition.

No significant changes in regional brain activation was found in the PT group as compared to the non-PT group under either condition. The current results may underestimate relevant differential effects of PT due to the lack of power caused by the small sample size. However, using a more lenient threshold, we did observe increased activation in the calcarine cortex, inferior temporal gyrus, and inferior occipital gyrus at Time 2 in the PT group, as compared with the non-PT group. These regions are known to be involved in processing facial information, including emotion recognition (Collins et al., 2012; Pitcher et al., 2011). Indeed, previous neuroimaging studies using RMET also reported involvement of these regions during this task (Adams et al., 2010; Castelli et al., 2010; Schmidt et al., 2020), which agrees with our findings. Furthermore, additional ROI analysis showed activation changes in overlapping region in the left visual cortex (including occipital fusiform gyrus). However, these regions did not survive correction for multiple testing, and the findings should be interpreted cautiously. Planned within-group comparisons showed significantly increased activation in the occipital part of the fusiform gyrus while judging others’ affective states from facial features only in the PT group under the ToM condition. The fusiform gyrus has been reported to be involved in face and object perception (Fusar-Poli et al., 2009), understanding of socioemotional meanings (Gallagher et al., 2000), and in the perception of emotions in facial expressions (Baron-Cohen et al., 1999; Greimel et al., 2010; Li et al., 2019). In one study, using the RMET, Batista et al. (2017) found a positive correlation between ToM scores and cortical thickness in the fusiform gyrus. In another study, Li et al. (2019) used intracranial electrodes and suggested that the fusiform gyrus participates in a relatively early stage of facial expression processing. Thus, we presumed that the increased brain activity and reduced reaction times seen in the PT group might reflect their improved sensitivity/efficiency to others’ affective states.

Taken together, we presumed that these changes might reflect that PT could reduce stress, and involvement of the fusiform gyrus. However, the causal relationship was not clarified in this study since this could not be proven by correlation analysis.

This study has some limitations. First, as mentioned above, it included a relatively small sample size and lacked a control group comprising mothers of typically developed children. Second, as all psychometric measures were self-reported, subjective bias might be present. Lastly, because of the single-center study design, the participants’ demographic variability might be limited. Future research should address these limitations.

## Conclusions

In summary, we examined the neural and behavioral effects of PT on mothers rearing children with ADHD. Mothers who participated in PT showed significantly reduced stress and improved parenting practices, and elevated activity in the fusiform gyrus during emotion recognition. This suggests that participation in PT can reduce stress, which may consequently increase activation of the fusiform gyrus.

## Supplementary information

Below is the link to the electronic supplementary material.

Supplementary file1 (DOCX 204 KB)

## Data Availability

The dataset used for the current study is not publicly available due to the Privacy Act of Japan. Regarding questions about this data, readers may contact Professor Akemi Tomoda (atomoda@u-fukui.ac.jp).
