# Supplementary material for: Neural and behavioral effects of parent training on emotion recognition in mothers rearing children with attention-deficit/hyperactivity disorder
Source: Brain Imaging Behav. 2023 Apr 20;17(4):436–49. doi: 10.1007/s11682-023-00771-9 (PMC10435396; doi:10.1007/s11682-023-00771-9)
Supplement: Supplementary file 1 — Supplementary file1 (DOCX 204 KB) [file 11682_2023_771_MOESM1_ESM.docx]

**Supplementary Material 1**

***Parent training***

In this study, we modified an existing parent training (PT) program (Iwasaka 2012) that was a version of an American parenting program (Barkley 1995; Whitham 1995) adapted for Japan, focusing on the management of children’s behavioral problems, including those related to attention-deficit/hyperactivity disorder (ADHD). Following the guidelines listed in the study by Iwasaka (2012), we reorganized our PT program to be dedicated to mothers of children with ADHD as a family support intervention. This PT program comprised 13 consecutive sessions and was conducted with a small group of candidates (3–5 people). PT was delivered in a lecture room located at the University of Fukui by two trained clinical psychologists. Each session lasted 2 h and sessions were held weekly. Thus, the course took approximately 90 days to complete. The 13 sessions were divided into five parts, in which mothers received training on the psychological/medical knowledge of ADHD and stress management skills (e.g., how to reduce stress) (sessions 1–3); training on how to observe and appropriately respond to the child’s behaviors and modify their parenting skills (e.g., praising the child’s good behavior) (sessions 4–6); training on how to provide clear explanations and rules for their children regarding their ADHD symptoms (e.g., getting the child's attention before giving instructions) (sessions 7–9); and training on effective methods to respond appropriately to the children’s non-adaptive behaviors stemming from their ADHD symptoms (e.g., how to ignore temper tantrums) (sessions 10–12); and a review session (session 13).

**Supplementary Material 2**

**Figure S1.** Example of a picture used during the trials.


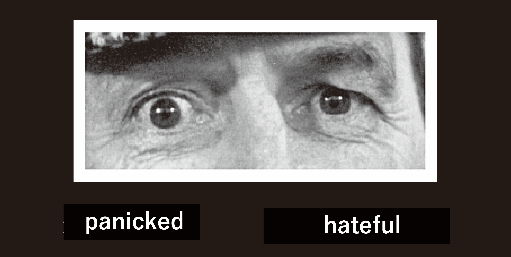

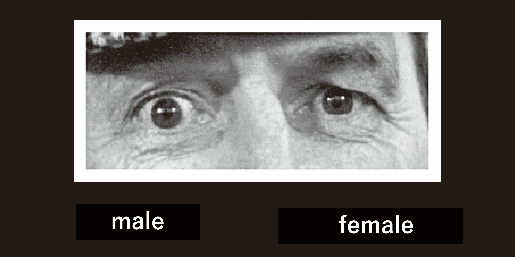


Example pictures used in training trials. The photo on the left was used under the ToM condition, and that on the right was used under the GeN condition.

Abbreviations: ToM, Theory of Mind; GeN, Gender judgement.

**Supplementary Material 3**

**Full results of 2 × 2 analyses of variance** **(Time [Time 1, Time 2] × Group [PT, non-PT]) conducted for questionnaire scores and Reading the Mind in the Eyes test (RMET) performance between Time 1 and Time 2.**

**Questionnaire scores**

BDI-II scores: Time F(1,21) = 1.952, *p* = 0.177; Group F(1,21) = 0.048, *p* = 0.829; Group x Time F(1,21) = 1.076, *p* = 0.311

PSI child domain scores: Time F(1,21) = 1.725, *p* = 0.203; Group F(1,21) = 0.036, *p* = 0.851; Group x Time F(1,21) = 10.076, *p* = 0.005

PSI parent domain scores: Time F(1,21) = 0.014, *p* = 0.907; Group F(1,21) = 0.052, *p* = 0.821; Group x Time F(1,21) = 8.804, *p* = 0.007

PS overreactivity scores: Time F(1,21) = 2.267, *p* = 0.117; Group F(1,21) = 1.826, *p* = 0.191; Group x Time F(1,21) = 4.611, *p* = 0.044

PS laxness scores: Time F(1,21) = 0.256, *p* = 0.618; Group F(1,21) = 0.067, *p* = 0.799; Group x Time F(1,21) = 0.146, *p* = 0.706

**RMET performance**

Reaction times for each condition

ToM condition: Time F(1,21) = 4.014, p = 0.058; Group F(1,21) = 0.391, p = 0.538; Group × Time F(1,21) = 4.323, p = 0.050 (0.050041)

GeN condition: Time F(1,21) = 1.660, p = 0.212; Group F(1,21) = 1.304, p = 0.266; Group × Time F(1,21) = 0.022, p = 0.884

Accuracy for each condition

ToM condition: Time F(1,21) = 3.857, p = 0.063; Group F(1,21) = 0.065, p = 0.801; Group × Time F(1,21) = 0.172, p = 0.682

GeN condition: Time F(1,21) = 1.615, p = 0.218; Group F(1,21) = 0.022, p = 0.882; Group × Time F(1,21) = 2.369, p = 0.139

Reaction times for only correct responses under the ToM condition

Time F(1,21) = 8.652, p = 0.008; Group F(1,21) = 0.333, p = 0.570; Group × Time F(1,21) = 4.229, p = 0.052

Reaction times for incorrect responses only under the ToM condition

(since one participant had a perfect score at Time 1, her data were omitted from the analysis as a missing value]

Time F(1,20) =0.115, p = 0.738; Group F(1,20) = 0.403, p = 0.533; Group × Time F(1,20) = 3.555, p = 0.074

Abbreviations:

PT, mothers enrolled in parent training intervention; non-PT, mothers who did not enroll in the parent training intervention; BDI-II, Beck Depression Inventory-II; PSI, Parenting Stress Index; PS, Parenting Scale; RMET, Reading the Mind in the Eyes Test; RTs, Reaction times (ms); Accuracy, correct rate (%); ToM, Theory of Mind condition; GeN, Gender judgement condition

**Supplementary Material 4**

**Table S1.** Regions showing between- and within-group differences ([PT/non-PT] vs. [Time 1/Time 2])

| Activation during ToM condition | | | | | | | |
| --- | --- | --- | --- | --- | --- | --- | --- |
| Anatomical region | Side | MNI coordinates | | | Cluster | Voxel | Cluster |
|  |  | x | y | z | Size | T value | P value |
| PT (Time 1 > Time 2) > non-PT (Time 1 > Time 2) | | | | | | | |
| [No significant activations] | | | | | | | |
| PT (Time 2 > Time 1) > non-PT (Time 2 > Time 1) | | | | | | | |
| Inferior temporal gyrus | Left | -45 | -51 | -15 | 61 | 4.60 | 0.67 |
| Calcarine cortex | Right | 18 | -81 | 0 | 60 | 4.46 | 0.68 |
| Calcarine cortex | Left | -12 | -90 | 0 | 75 | 3.89 | 0.51 |
| Inferior occipital gyrus | Right | 48 | -66 | -3 | 53 | 3.50 | 0.76 |
| Activation during GeN condition | | | | | | | |
| Anatomical region | Side | MNI coordinates | | | Cluster | Voxel | Cluster |
|  |  | x | y | z | Size | T value | P value |
| PT (Time 1 > Time 2) > non-PT (Time 1 > Time 2) | | | | | | | |
| [No significant activations] | | | | | | | |
| PT (Time 2 > Time 1) > non-PT (Time 2 > Time 1) | | | | | | | |
| [No significant activations] | | | | | | | |

Note: The threshold was set at *p* < 0.005, uncorrected at the peak level with an extent of < 40 voxels. Locations were deﬁned using the SPM neuromorphometrics atlas.

Abbreviations: MNI, Montreal Neurologic Institute; PT, mothers enrolled in parent training intervention; non-PT, mothers who did not enroll in the parent training intervention; ToM, Theory of Mind; GeN, Gender judgement.

**Supplementary Material 5**

**Table S2.** Results of the ROI analysis showing between- and within-group differences ([PT/non-PT] vs. [Time 1/Time 2])

| Activation during ToM condition | | | | | | | |
| --- | --- | --- | --- | --- | --- | --- | --- |
| Anatomical region | Side | MNI coordinates | | | Cluster | Voxel | Cluster |
|  |  | x | y | z | Size | T value | P value |
| PT (Time 1 > Time 2) > non-PT (Time 1 > Time 2) | | | | | | | |
| [No significant activations] | | | | | | | |
| PT (Time 2 > Time 1) > non-PT (Time 2 > Time 1) | | | | | | | |
| Calcarine cortex | Left | -15 | -90 | -3 | 42 | 3.73 | 0.46 |
|  |  |  |  |  |  |  |  |
|  |  |  |  |  |  |  |  |
|  |  |  |  |  |  |  |  |
| Activation during GeN condition | | | | | | | |
| Anatomical region | Side | MNI coordinates | | | Cluster | Voxel | Cluster |
|  |  | x | y | z | Size | T value | P value |
| PT (Time 1 > Time 2) > non-PT (Time 1 > Time 2) | | | | | | | |
| [No significant activations] | | | | | | | |
| PT (Time 2 > Time 1) > non-PT (Time 2 > Time 1) | | | | | | | |
| [No significant activations] | | | | | | | |

Note: The threshold was set at *p* < 0.005, uncorrected at the peak level with an extent of < 40 voxels. Locations were deﬁned using the SPM neuromorphometrics atlas.

Abbreviations: MNI, Montreal Neurologic Institute; PT, mothers enrolled in parent training intervention; non-PT, mothers who did not enroll in the parent training intervention; ToM, Theory of Mind; GeN, Gender judgement.

**Supplementary Material 6**

**Table S3. Associations between changes of activation in occipital fusiform gyrus, and RTs and questionnaire scores**

|  | Activation  (left OFG) | PSI  (Child domain) | PSI  (Parent domain) | PS  (Overreactivity) | RTs |
| --- | --- | --- | --- | --- | --- |
| Activation  (left OFG) |  |  |  |  |  |
| PSI  (Child domain) | *r* = 0.184,  *p* = 0.548 |  |  |  |  |
| PSI  (Parent domain) | *r* = −0.273,  *p* = 0.367 | r = 0.173,  p = 0.571 |  |  |  |
| PS  (Overreactivity) | *r* = 0.248,  *p* = 0.415 | *r* = 0.271,  *p* = 0.370 | *r* = −0.343,  *p* = 0.032 |  |  |
| RTs | *r* = −0.098,  *p* = 0.749 | r = -0.041, p = 0.895 | r = −0.539, p = 0.057 | *r* = 0.182,  *p* = 0.552 |  |

**Changes in values/scores are calculated by subtracting Time 1 from Time 2 (Time 2 – Time 1).**

Abbreviations:

PS, Parenting Scale; PSI, Parenting Stress Index; P RTs, Reaction Times (ms); OFG, occipital fusiform gyrus

**References**

Barkley, R. (1995). *Taking charge of ADHD: The complete authoritative guide for parents*. Boystown, NE: The Guilford Press

Iwasaka, H. Ed. (2012). *Pearento Torêningu Gaidobukku (Parent Training Guidebook)*. Tokyo, Japan: Jihou Inc. (in Japanese)

Whitham, C. (1995). *Win the whining war and other skirmishes: A family peace plan*. Pasadena, CA: Perspective Pub.
